# Transitioning to Omnipod 5^®^: Effectiveness, Safety, and Patient-Reported Outcomes of a Tubeless Automated Insulin Delivery System in Adults with Type 1 Diabetes Mellitus

**DOI:** 10.3390/biomedicines14051136

**Published:** 2026-05-17

**Authors:** Carmelo Gusmano, Rossella Cannarella, Concetta Finocchiaro, Gianfranco Gruttadauria, Rosario Randazzo, Rosita A. Condorelli, Sandro La Vignera, Aldo E. Calogero, Giuseppe Papa

**Affiliations:** 1 Unit of Metabolic and Endocrine Disease, “Centro Catanese di Medicina e Chirurgia” Clinic, 95126 Catania, Italy; carmelo.gusmano@yahoo.it (C.G.); cettyfinocchiaro@iol.it (C.F.); gpapa_98@yahoo.com (G.P.); 2 Department of Clinical and Experimental Medicine, University of Catania, Via S. Sofia 78, 95123 Catania, Italy; rosario.randazzo1996@gmail.com (R.R.); rosita.condorelli@unict.it (R.A.C.); sandrolavignera@unict.it (S.L.V.);; 3 Unit of Diabetology, Caltanissetta Health District, 93100 Caltanissetta, Italy

**Keywords:** type 1 diabetes mellitus, automated insulin delivery, Omnipod 5^®^, patient-reported outcomes

## Abstract

**Background and Aims:** Automated insulin delivery (AID) systems are standard of care for type 1 diabetes mellitus (T1DM). Tubeless AID systems may improve treatment acceptance, but real-world European data in patients transitioning from multiple daily injections (MDI) or open-loop patch pump therapy are limited. This study evaluated real-world glycemic, safety, and quality-of-life (QoL) outcomes after transition to a tubeless automated closed-loop system (Omnipod 5^®^, OP5^®^). **Research Design and Methods**: In this prospective, multicenter observational study, adults with T1DM transitioned from MDI or open-loop continuous subcutaneous insulin infusion to OP5^®^ and were followed for 180 days. Continuous glucose monitoring-derived metrics and validated patient-reported outcome measures were assessed. Subgroup analyses were performed by prior therapy. **Results**: Of the 94 enrolled participants, 88 completed the study. At 180 days, HbA1c decreased from 7.5% to 7.1% (*p* < 0.001), and time in range increased from 59.0% to 68.0% (*p* < 0.001) without increased hypoglycemia. The proportion achieving TIR_70–180_ ≥ 70% rose from 12.5% to 43.2%. Improvements were greater among prior MDI users. Treatment satisfaction and diabetes-related QoL improved significantly. The mean time in automated mode was 90.9%. **Conclusions**. Transition to tubeless AID significantly improved glycemic and psychosocial outcomes, supporting its effectiveness in routine clinical practice.

## 1. Introduction

Automated insulin delivery (AID) systems, which combine continuous glucose monitoring (CGM) data with adaptive control algorithms, have substantially advanced the management of patients with type 1 diabetes mellitus (T1DM) by enabling real-time optimization of insulin delivery and reducing the burden of manual diabetes self-management [1,2,3,4,5,6]. A growing body of robust evidence demonstrating improvements in glycemic outcomes, safety, and quality of life (QoL) has led to the endorsement of AID systems as the standard of care for patients with T1DM in major international clinical practice guidelines [7,8].

Beyond algorithmic performance, device miniaturization and the availability of tubeless insulin delivery represent clinically relevant advances, as long-term adherence is strongly influenced by wearability, comfort, and the impact of medical devices on body image and daily activities [9,10].

The Omnipod 5^®^ OP5^®^ system is the first tubeless patch pump incorporating a hybrid closed-loop (HCL) insulin delivery system. It incorporates a predictive control algorithm (SmartAdjust^TM^) embedded within the pod, which processes CGM data and autonomously modulates insulin basal rate administration according to customizable glucose targets (110–150 mg/dL) that can be tailored to different time segments throughout the day. On the other hand, meal-related boluses still require user input, consistent with other commercially available HCL systems [11]. This distinguishes HCL systems from fully closed-loop systems, which aim to automate both basal and bolus insulin delivery, although such systems are not yet widely available in routine clinical practice.

Retrospective, prospective, and real-world studies have demonstrated the effectiveness, safety, and favorable impact of OP5^®^ on patient-reported outcome measures (PROMs) [6,12,13,14,15]; however, evidence from European cohorts remains relatively limited. Differences in healthcare organization, reimbursement policies, cultural factors, and patient characteristics may influence both the adoption and real-world performance of AID technologies across countries.

Accordingly, the present study aimed to evaluate, in a multicenter Italian cohort, the effects of transitioning from conventional therapy, either multiple daily injections (MDIs) or open-loop continuous subcutaneous insulin infusion (CSII), to OP5^®^ on glycemic control, safety, and QoL. Notably, all participants previously treated with open-loop CSII were users of the Omnipod DASH^®^ (OPD^®^) system, which does not incorporate an AID algorithm. Importantly, this study addresses a clinically relevant gap by evaluating the incremental benefit of automation in patients already using a tubeless patch pump system (Omnipod DASH^®^), a population that has been scarcely investigated in previous studies.

## 2. Materials and Methods

### 2.1. Ethics Approval

The study was conducted at three anti-diabetes centers in Sicily in accordance with the principles of the Declaration of Helsinki. The study protocol was approved by the ethics committees of the participating institutions, and written informed consent for the use of clinical and biochemical data was obtained from all participants.

### 2.2. Study Protocol

Patient enrollment was conducted between December 2024 and June 2025, allowing for the completion of the 180-day follow-up for all participants by December 2025. A total of 94 adults with T1DM were consecutively enrolled; participants had been previously treated with either MDI or open-loop CSII. Among those using open-loop CSII, eligibility was restricted to patients previously treated with the OPD^®^ system, which is not equipped with an AID algorithm, in combination with Dexcom G6™ or G7™ (DexCom, Inc., San Diego, CA, USA) or FreeStyle Libre 2 plus sensors (Abbott Diabetes Care Inc., Alameda, CA, USA). This criterion was applied to ensure a more homogeneous study population and to minimize variability related to prior insulin delivery technology. Inclusion criteria comprised a diagnosis of T1DM for at least one year, CGM use for a minimum of three months prior to enrollment, and willingness to transition to the OP5^®^ system (for participants previously using OPD^®^) (Insulet Corporation, Acton, MA, USA). Exclusion criteria included pregnancy and the presence of severe comorbid conditions, such as significant cardiac and/or pulmonary disease, stage III or higher chronic renal failure, active malignancy, or chronic glucocorticoid therapy.

All patients completed four structured training sessions, each lasting approximately two hours, before transitioning to the OP5^®^ system. The training program covered all components of OP5^®^, including the patch pump (POD), the controller, and the CGM devices (Dexcom G6^TM^ or G7^TM^ or FreeStyle Libre 2 Plus). Patients received instruction on bolus delivery, interpretation of CGM trend data, and overall system operation. Additional sessions focused on CGM sensor management, including sensor application, calibration when required, and interpretation of glucose trends, as well as on the AID system features, such as basal rate automation, customizable glucose targets, and management during physical activity. Patients were also educated on carbohydrate counting, insulin-to-carbohydrate ratio (I/CHO), correction factor (insulin sensitivity), glycemic targets, and active insulin time. Training included programming of basal and temporary basal rates in manual mode. Pump and sensor data were automatically uploaded via the controller to the Glooko^TM^ platform for review by the clinical care team.

At the baseline visit, insulin pump settings were established for all patients, including basal insulin rates, insulin sensitivity factor (ISF), and I/CHO ratio. The initial glucose target was set at 110 mg/dL in all patients; by default, an active insulin time of 3 h and a correction threshold of 120 mg/dL were also programmed. After four weeks of system use, the glucose target could be increased to 120 or 130 mg/dL, either for the entire 24 h period or for specific time intervals, based on ambulatory glucose profile (AGP) data and individual risk of hypoglycemia.

### 2.3. Study Endpoints

At baseline, anthropometric measurements, insulin delivery parameters, laboratory-measured HbA1c, and CGM data from the 14 days preceding the visit were collected during previous therapy. Follow-up evaluations were performed at 90 and 180 days after initiation of the OP5^®^ system, with CGM data referring to the 14 days preceding each follow-up visit. At the final visit (180 days), anthropometric measurements were reassessed, and HbA1c was measured again. Baseline CGM data were downloaded from the respective manufacturers’ proprietary platforms, whereas integrated pump–sensor data at the 90-day and 180-day visits were downloaded from the Glooko^TM^ platform. QoL questionnaires were administered at baseline and repeated at the final visit (180 days).

The primary endpoints included glycemic control, safety, and QoL. Glycemic outcomes comprised time in range (TIR_70–180_), time above range (TAR_181–250_ and TAR_>250_), HbA1c (%), glucose management indicator (GMI, %), and glycemic variability, expressed as the coefficient of variation (CV, %). Safety endpoints included time below range (TBR_54–69_ and TBR_<54_) and the presence of diabetic ketoacidosis (DKA). Baseline CGM data were obtained from manufacturer-specific platforms, whereas follow-up data were extracted from the Glooko™ platform. Although these systems provide standardized and broadly comparable AGP-derived metrics, differences in proprietary algorithms, calibration approaches, and data processing may introduce measurement variability and potentially affect the magnitude of observed changes over time.

PROMs included the Diabetes Treatment Satisfaction Questionnaire (DTSQ), the CSII-QoL questionnaire, the Fear of Hypoglycemia scale (FH-15), the Problem Areas in Diabetes short form (PAID-5), and the WHO-5 Well-Being Index (WHO-5 WBI).

### 2.4. Statistical Analysis

Continuous variables are presented as mean ± SD when normally distributed and as median (interquartile range, IQR) when non-normally distributed. Effect sizes (e.g., mean differences and 95% confidence intervals) were reported where appropriate. Normality was assessed using the Shapiro–Wilk test. Between-group comparisons at enrollment according to previous insulin delivery modality (MDI or open-loop CSII OPD^®^) were performed using the unpaired Student’s *t* test or the Mann–Whitney U test, as appropriate. Within-group comparisons at different time points were analyzed using the Wilcoxon signed-rank test (for two time points) or the Friedman test (for three time points). The same statistical approach was applied to compare questionnaire scores between baseline and 180 days.

Changes in metabolic and CGM-derived endpoints (HbA1c, mean glucose, GMI, CV, TIR_70–180_, TAR_181–250_, TAR_>250_, TBR_40–54_, and TBR_<54_) across time points (baseline, 90 days, and 180 days) were analyzed using one-way analysis of variance (ANOVA) or the Kruskal–Wallis test, according to data distribution. Homogeneity of variances was assessed using Levene’s test. No formal adjustment for multiple comparisons was performed. Therefore, all statistical findings, particularly subgroup analyses and secondary endpoints, should be interpreted as exploratory and hypothesis-generating rather than confirmatory. The proportions of participants achieving guideline-recommended glycemic targets (TIR_70–180_ > 70%, TAR_181–250_ < 25%, TAR_>250_ < 5%, TBR_40–54_ < 4%, and TBR_<54_ < 1%) were compared using the χ^2^ test.

To assess the impact of previous insulin delivery modality on outcomes over time, a two-way ANOVA with Tukey’s post hoc multiple-comparison test was performed.

No formal a priori sample size calculation was performed due to the observational design. However, based on previously reported improvements in TIR_70–180_ of approximately 9–10% with Omnipod 5^®^, the present sample size can be considered adequate to detect clinically meaningful changes. Finally, the CSII-QoL questionnaire, originally developed for insulin pump users, was administered to all participants; however, its applicability to patients transitioning from MDI may be limited.

All statistical analyses were conducted using MedCalc Software Ltd. (Ostend, Belgium), version 19.6 (64-bit). A two-sided *p* value < 0.05 was considered statistically significant.

## 3. Results

### 3.1. General and Metabolic Characteristics of the Patient Cohort at Enrollment

A total of 94 adults with T1DM were enrolled, of whom 88 completed the study protocol. Two participants discontinued the intervention and reverted to MDIs due to unwillingness to continue insulin pump therapy, and four were lost to follow-up. These six patients were excluded from both baseline characterization and final data analysis (Supplementary Figure S1). Baseline characteristics of the six excluded participants (2 who discontinued the intervention and 4 lost to follow-up) were comparable to those of participants who completed the study in terms of age, diabetes duration, and baseline glycemic control. Due to the small number of excluded individuals, no formal statistical comparison was performed.

At study completion, 77 patients had set a glucose target of 110 mg/dL (87.5%), while 11 (12.5%) selected a target of 120–130 mg/dL. Most participants used the Dexcom^TM^ G6 continuous glucose monitoring system sensor (88.6%), whereas only 11.4% used either the FreeStyle Libre 2 Plus or Dexcom™ G7 sensors.

Approximately 30% of participants transitioned to OP5^®^ therapy from MDI, while the remaining 70% switched from OPD^®^. Their baseline demographic and metabolic characteristics are summarized in Table 1. The median age of participants was 38.5 years (range 18–67 years), and the mean duration of diabetes was approximately 17 years. At enrollment, overall glycemic control was suboptimal, with a mean HbA1c of 7.6%, consistent with CGM-derived metrics (GMI 7.5%) and a CV at the upper limit of the recommended range (39.0%). All CGM parameters reflecting hyperglycemic exposure, TIR_70–180_, TAR_180–250_, and TAR_>250_, were outside the established targets at baseline. Notably, participants previously treated with MDI exhibited higher HbA1c, mean glucose, and GMI values, along with lower TIR_70–180_ and higher TAR_180–250_, compared with those previously using OPD^®^.

Student’s *t* test for independent samples or the Mann–Whitney U test was applied for normally and non-normally distributed variables, respectively.

### 3.2. Before–After Analysis

**Figure 1:**
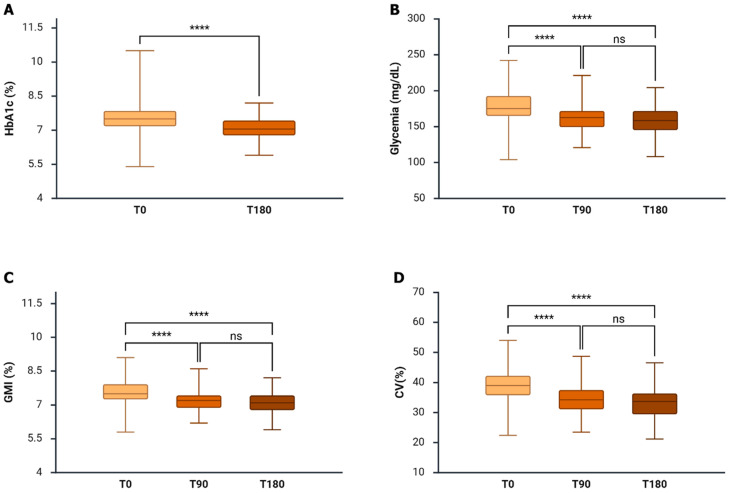
Effects of the Omnipod 5^®^ (OP5^®^) system on metabolic parameters. Transition to OP5^®^ was associated with significant reductions in HbA1c at 180 days (Panel (**A**)), mean glucose levels at 90 and 180 days (Panel (**B**)), and GMI at 90 and 180 days (Panel (**C**)), compared with baseline therapy. Glycemic variability, assessed by coefficient of variation (CV), was also significantly reduced from 90 days onward (Panel (**D**)). Data are presented as median (IQR) or mean ± SD, as appropriate. Abbreviations: GMI, glucose management indicator; CV, coefficient of variation. **** *p* < 0.0001.

Laboratory-measured HbA1_c_ decreased significantly from 7.5% (7.2–7.9%) at baseline to 7.1% (6.8–7.4%) at 180 days (*p* < 0.001), corresponding to a relative treatment effect of –0.4% (95% CI −0.6, −0.4) (Figure 1A). Mean glucose levels declined progressively from 175.2 mg/dL (163.7–191.9 mg/dL) at baseline to 162.6 (150.1–171.0 mg/dL) mg/dL at 90 days and 158.4 (145.9–171.0 mg/dL) mg/dL at 180 days (*p* < 0.001). Consistent reductions were observed in GMI, which decreased from 7.5% (7.3–7.9%) at baseline to 7.2% (6.9–7.4%) at 90 days and 7.1% (6.8–7.4%) at 180 days (Figure 1B,C). Glycemic variability, assessed by CV, was significantly reduced from 39.0 ± 5.4% at baseline to 34.2 ± 5.3% at 90 days and 33.3 ± 5.2% at 180 days (*p* < 0.001) (Figure 1D).

TIR_70–180_ increased from 59.0% (51.5–67.5%) at baseline to 69.0% (61.0–76.0%) at 90 days and 68.0 (61.0–79.0%) at 180 days (*p* < 0.001).

**Figure 2:**
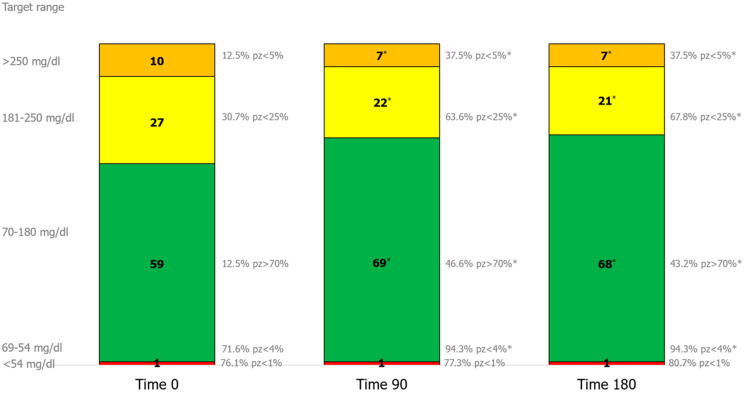
Effects of Omnipod 5^®^ (OP5^®^) on CGM-derived time-in-range metrics. Use of OP5^®^ was associated with significant increases in TIR_70–180_ and reductions in TAR_181–250_ and TAR_>250_ at both 90 and 180 days compared with baseline therapy. TBR_54–69_ and TBR_<54_ remained stable throughout follow-up. Data are expressed as median (IQR). * *p* < 0.05 versus baseline.

Notably, despite the low proportion of participants achieving target TIR_70–180_ at baseline (12.5%), 43.2% attained a TIR_70–180_ > 70% at 180 days, representing a 3.5-fold increase relative to enrollment (*p* < 0.0001) and consistent with international recommendations (Figure 2).

TAR decreased significantly for both moderate (180–250 mg/dL) and severe hyperglycemia (>250 mg/dL) at 90 and 180 days (*p* < 0.001 for all comparisons). By study completion, 67.8% and 37.5% of participants achieved the guideline-recommended targets for TAR_180–250_ (<25%) and TAR_>250_ (<5%), respectively, compared with 30.7% and 12.5% at baseline (Figure 2).

TBR_54–69_ and TBR_<54_ remained stable throughout the follow-up period, with no evidence of increased hypoglycemia. At study completion, 94.3% and 80.7% of participants met recommended targets for TBR_54–69_ (<4%) and TBR_<54_ (<1%), respectively (Figure 2).

### 3.3. Subgroup Analysis According to Prior Insulin Therapy (MDI or Open-Loop CSII OPD^®^)

A prespecified subgroup analysis assessed the effects of closed-loop insulin delivery on glycemic outcomes over 180 days according to prior insulin delivery modality (MDI or open-loop CSII OPD^®^).

Among participants previously treated with MDI, initiation of closed-loop therapy was associated with significant improvements in glycemic control. HbA1c decreased from 7.8% at baseline to 7.2% at 180 days (*p* < 0.0001). Mean glucose, GMI, and glycemic variability, assessed by CV, were significantly reduced at both 90 and 180 days (all *p* ≤ 0.006). TIR_70–180_ increased from 54% to 66% (*p* = 0.0001), primarily driven by reductions in TAR, while TBR remained unchanged.

Similarly, among participants previously treated with CSII OPD^®^, the initiation of closed-loop therapy was associated with a significant reduction in HbA1c (7.4% to 7.0%; *p* = 0.0035), mean glucose, GMI, and CV (all *p* ≤ 0.003). TIR_70–180_ increased from 61% to 72% at follow-up (*p* < 0.0001), accompanied by significant reductions in both moderate and severe hyperglycemias (TAR_180–250_ and TAR_>250_). A modest reduction in TBR_54–69_ was observed, whereas TBR_<54_ remained negligible (Table 2).

Overall, closed-loop therapy significantly improved CGM-derived glycemic outcomes irrespective of prior insulin delivery modality, with sustained reductions in hyperglycemia and glycemic variability and no increase in clinically relevant hypoglycemia.

### 3.4. Weight and Body Mass Index

No significant changes were observed in body weight (from 72.8 ± 13.4 kg to 73.2 ± 11.6 kg, *p* = 0.185), body mass index [from 25.5 (23.0–29.0) kg/m^2^ to 26.3 (23.0–29.0) kg/m^2^, *p* = 0.841], or waist circumference [from 91.0 (85.0–96.5.0) cm to 92.5 (86.0–98.5) cm, *p* = 0.346] over the study period.

### 3.5. Compliance with Therapy

At the 180-day follow-up, T1DM patients spent 90.9 ± 16.7% of the time in SmartAdjust^TM^ (Auto Mode), indicating high system adherence and strong acceptance of the automated insulin delivery algorithm.

### 3.6. Quality of Life and Psychosocial Outcomes

**Figure 3:**
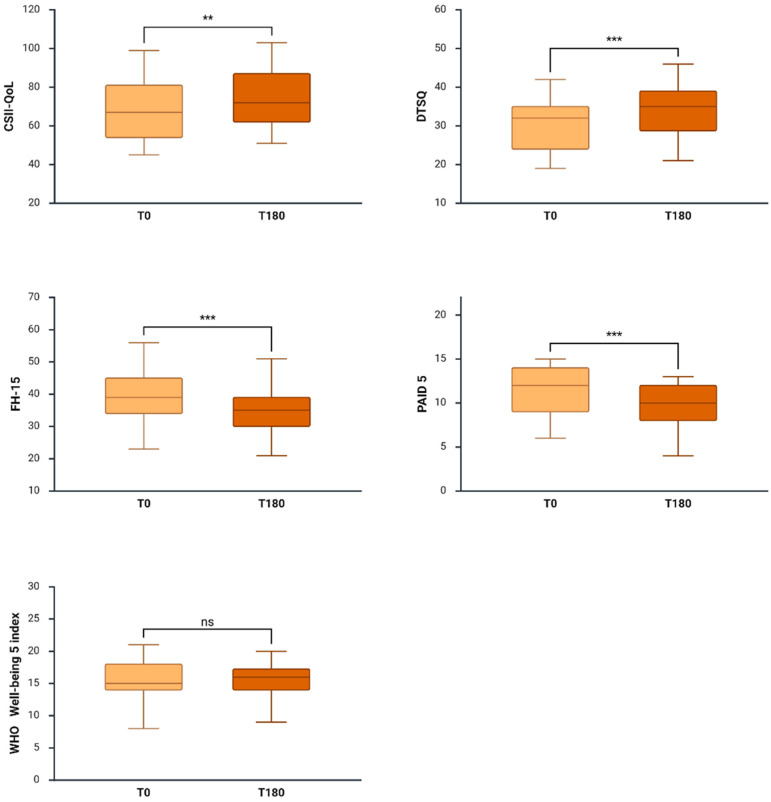
Effects of the Omnipod 5^®^ (OP5^®^) system on patient-reported outcome measures. Transition to OP5^®^ was associated with significant improvements in treatment satisfaction (DTSQ), CSII-related quality of life (CSII-QoL), fear of hypoglycemia (FH-15), and diabetes-related distress (PAID-5). No significant changes were observed in WHO-5 Well-Being Index scores. Data are presented as median (IQR). ** *p* < 0.01; *** *p* < 0.001 versus baseline.

Significant improvements were observed in CSII-QoL and treatment satisfaction (DTSQ), along with a reduction in fear of hypoglycemia (FH-15) and diabetes-related distress (PAID-5). Scores on the WHO-5 WBI did not significantly change over the study period (Figure 3). To account for the possible bias introduced by CSII-QoL use in MDI users, data were stratified according to previous MDI or OPD^®^ use. No differences were found at baseline and at the 3-month follow-up (Supplementary Figure S2).

### 3.7. Severe Hypoglycemia and Diabetic Ketoacidosis

No episodes of DKA or severe hypoglycemia requiring third-party assistance were reported during the study period.

## 4. Discussion

This multicenter real-world study demonstrates that transitioning from conventional insulin therapy, either MDI or open-loop CSII OPD^®^, to the tubeless AID system OP5^®^ results in clinically meaningful improvements in both metabolic control and psychosocial outcomes in adults with T1DM. Importantly, this study provides real-world evidence from a European cohort and adds to the existing literature by specifically evaluating the combined metabolic and QoL effects of transitioning from either MDI or a non-automated patch pump to a fully automated tubeless system. However, the study population was geographically restricted to Sicily, and healthcare system factors (e.g., training, reimbursement, patient selection) may limit generalizability to other settings.

From a metabolic perspective, OP5^®^ use was associated with significant increases in TIR_70–180_, reductions in HbA1c and glycemic variability, and sustained decreases in hyperglycemic exposure, without an increase in clinically relevant hypoglycemia. At 180 days, mean TIR_70–180_ increased by 11.5%, corresponding to approximately 2.4 additional hours per day spent within the target glucose range. This magnitude of improvement is consistent with findings from the OP5^®^ pivotal trial and large real-world studies, in which TIR_70–180_ values of approximately 68–70% were reported after system initiation [12].

Larger improvements in TIR_70–180_ have been reported in some randomized controlled trials (RCTs) and observational studies. In a recent RCT comparing OP5^®^ with sensor-augmented pump therapy, participants treated with OP5^®^ (n = 132) experienced a TIR_70–180_ increase of 17.3% (approximately 4.2 h/day). However, baseline glycemic control in that study was significantly poorer than in the present cohort, with higher baseline HbA1c (8.5%) and lower baseline TIR_70–180_ (45.5 ± 14.8%) [16]. Similarly, an observational study involving 353 patients reported an approximate 16% increase in TIR_70–180_, again in the context of substantially lower baseline TIR_70–180_ values (48%) compared with the patients enrolled in the present study [17]. In a prospective observational study of pediatric and adolescent patients transitioning from OPD^®^ to OP5^®^, TIR_70–180_ increased from 55.1% at baseline to 69.0% after only 28 days of observation [18]. These findings are consistent with our results in participants transitioning from OPD^®^, who demonstrated an increase in TIR_70–180_ from 59.6% to 70.4% at 180 days.

Differences in the magnitude of TIR_70–180_ improvement across studies likely reflect variations in baseline glycemic control, as greater gains are typically observed in populations with poorer baseline metrics. Consistent with this interpretation, the largest improvements were observed among participants transitioning from MDI, who exhibited higher baseline HbA1c and lower TIR_70–180_. Although statistically significant, the magnitude of HbA1c reduction was modest and should be interpreted cautiously in terms of long-term clinical impact. In contrast, improvements in TIR_70–180_ may be more clinically meaningful, given their established association with complication risk [19,20]. Nevertheless, patients switching from open-loop patch pump therapy also experienced clinically meaningful benefits. Notably, participants previously using OPD^®^ achieved substantial improvements in glycemic outcomes despite prior familiarity with patch pump technology, highlighting the incremental value of automation beyond the insulin delivery modality alone. However, these findings may reinforce the effectiveness of OP5^®^ across a broad spectrum of prior treatment experiences. In addition, the absence of correction for multiple testing increases the risk of type I error [21] and further supports a cautious interpretation of secondary analyses. Baseline imbalances may have influenced the magnitude of the observed changes.

Regarding longer-term glycemic control, we observed a mean reduction in HbA1c of 0.4% at 180 days. This result aligns with data from a large real-world study involving 2504 individuals transitioning to OP5^®^ [22], as well as with the findings from the pivotal trial, including participants aged 6 to 70 years [12]. As expected, the greatest improvements in glycemic outcomes were observed in participants previously treated with MDI. However, clinically meaningful improvements were also evident among those transitioning from open-loop pump therapy, indicating that OP5^®^ confers benefits even in patients already experienced with insulin pump use.

In parallel with improvements in CGM-derived metrics, OP5^®^ use was associated with significant benefits in patient-reported outcomes. Treatment satisfaction, pump-related QoL, fear of hypoglycemia, and diabetes-related distress all improved after 180 days of AID therapy. Although significant improvements were observed in several patient-reported outcomes, these findings should be interpreted with caution due to the absence of a control group and the potential influence of expectation or placebo effects [23]. The lack of change in the WHO-5 Well-Being Index score may be explained by its generic nature, as its items are not specifically designed to capture diabetes- or device-related benefits, which may therefore remain undetected despite improvements associated with AID system use.

High adherence to automated mode use further supports strong patient acceptance and trust in the OP5^®^ algorithm. This observation is clinically relevant, as a sustained engagement with AID systems is essential for achieving long-term metabolic benefits [24]. Although all participants received structured education before system initiation, consistent with best clinical practice, the high levels of adherence and satisfaction observed suggest that the tubeless automated system was well integrated into daily life, even among individuals previously accustomed to different insulin delivery modalities.

Concerning safety outcomes, the absence of severe hypoglycemia and DKA should be interpreted cautiously, given the limited sample size and duration of follow-up. Device-related adverse events were not systematically observed in our cohort, as local skin reactions were rare and mild, and technical issues involving POD and PDM were virtually absent.

The strengths of this study include its multicenter design, real-world setting, comprehensive assessment of both metabolic and psychosocial outcomes, and the relatively homogeneous cohort transitioning from conventional therapy. Limitations include the modest sample size, the absence of a randomized control group, and the relatively short duration of follow-up. The before–after design without a control group represents a major limitation, as improvements may be partially explained by regression to the mean and by behavioral changes following structured education and increased clinical attention at study initiation [25]. Therefore, causality cannot be established, and the observed associations should be interpreted with caution. Although participants were managed within a structured clinical setting with standardized training—consistent with current guideline recommendations for AID system initiation—this approach may have contributed to higher adherence and improved clinical outcomes and thus may limit the generalizability of our findings to less structured care settings [26]. From a statistical perspective, the use of one-way ANOVA for repeated measures represents a limitation, as more appropriate longitudinal models (e.g., repeated-measures ANOVA or mixed-effects models) [27] could better account for within-subject correlations. A small number of participants were excluded due to discontinuation or loss to follow-up. Although their baseline characteristics were broadly comparable to those of participants who completed the study, attrition bias cannot be excluded. If these individuals had lower adherence or less favorable outcomes, their exclusion may have led to a slight overestimation of treatment effects. Also, the use of the CSII-QoL questionnaire in participants without prior pump experience may have introduced bias, including potential floor effects [28], although no differences were found after stratification of CSII-QoL data based on MDI or OPD^®^ use. Finally, the use of different CGM data platforms at baseline and follow-up may have introduced measurement variability due to differences in proprietary algorithms, calibration procedures, and AGP metric calculation methods [29]. This may have slightly influenced the magnitude of observed longitudinal changes in CGM-derived outcomes.

Future studies involving larger populations, longer observation periods, and controlled designs are warranted to confirm these findings and to evaluate the durability of psychosocial benefits over time.

## 5. Conclusions

In adults with T1DM, transition to a tubeless AID system is associated with significant and sustained improvements in glycemic control, including increased TIR_70–180_, reduced hyperglycemia, and lower glycemic variability, without increasing hypoglycemia risk. These metabolic benefits are accompanied by meaningful improvements in treatment satisfaction, QoL, and diabetes-related emotional burden, supporting high acceptance and adherence to therapy [30]. Collectively, these findings may support the integration of tubeless closed-loop technology into routine clinical practice and emphasize the importance of addressing both metabolic and psychosocial dimensions of diabetes management. Beyond confirming previous evidence, the present study provides clinically relevant insights into the incremental value of automation in patients already familiar with patch pump therapy and real-world implementation in European healthcare settings.

## Figures and Tables

**Table 1:** General characteristics and metabolic parameters of the study cohort: all subjects, MDI group, and CSII OPD^®^ group.

**General Characteristics**				
	**All Patients**	**MDI Group**	**CSII OPD** ^® ^ **Group**	***p*** **-Value**
n	88	31	57	-
Age (years)	38.5 (28.5–53.0)	33.0 (23.3–49.0)	45.0 (31.0–53.3)	0.0371
Male (n, %)	52 (59.1%)	21 (67.7%)	31 (54.4%)	0.2262
Duration of the disease (years)	16.5 (9.0–28.0)	14.5 (5.5–19.5)	18.5 (10.0–28.0)	0.1545
Weight (kg)	72.8 ± 13.4	74.6 ± 14.1	71.8 ± 13.0	0.3480
BMI (kg/m^2^)	25.6 (23.0–29.0)	26.0 (23.2–30.7)	25.3 (22.6–28.2)	0.2180
**Metabolic Parameters**				
	**All Patients**	**MDI Group**	**CSII OPD^®^ Group**	***p *** **Value**
HbA1c (%)	7.5 (7.2–7.9)	7.8 (7.4–8.3)	7.4 (7.2–7.7)	0.0009
Glucose (mg/dL)	175.2 (163.7–191.9)	187.7 (172.1–200.3)	175.2 (161.6–179.3)	0.0021
GMI (%)	7.5 (7.3–7.9)	7.8 (7.4–8.1)	7.5 (7.2–7.6)	0.0021
CV (%)	39.0 ± 5.4	39.4 ± 4.7	38.5 ± 5.7	0.4450
TIR_70–180_ (%)	59.0 (51.5–67.5)	54.0 (46.0–61.0)	61.0 (54.0–69.0)	0.0084
TAR_180–250_ (%)	27.0 (23.0–31.0)	31.0 (24.5–33.0)	26.0 (22.5–29.0)	0.0082
TAR_>250_ (%)	10.0 (7.0–15.0)	12.0 (7.0–19.3)	10.0 (6.8–12.5)	0.1356
TBR_54–69_ (%)	1.0 (0.0–4.0)	1.0 (0.0–3.0)	2.0 (0.8–4.0)	0.2811
TBR_<54_ (%)	0.0 (0–0)	0.0 (0–0)	0.0 (0–1.0)	0.4862

Abbreviations. BMI, body mass index; CSII, continuous subcutaneous insulin infusion; CV, coefficient of variation; GMI, glucose management index; F, female; MDIs, multiple daily injections; OPD^®^, Omnipod Dash^®^; TAR, time above range; TBR, time below range; TIR, time in range.

**Table 2:** Effects of the closed-loop insulin delivery system Omnipod 5^®^ on metabolic parameters and time in target ranges: subgroup analysis according to previous therapy (MDI or open-loop continuous subcutaneous insulin infusion (CSII) Omnipod dash^®^ (OPD^®^)).

Metabolic Parameters	MDI Group					CSII OPD^®^ Group				
	T0	T90	T180	Adj *p* Value (T90 vs. T0)	Adj *p* Value (T180 vs. T0)	T0	T90	T180	Adj *p* Value (T90 vs. T0)	Adj *p* Value (T180 vs. T0)
HbA1c (%)	7.8 (7.4–8.3)	-	7.2 (7.0–7.5)	-	<0.0001	7.4 (7.2–7.7)	**-**	7.0 (6.8–7.4)	-	0.0035
Glucose (mg/dL)	187.7 (172.1–200.3)	166.8 (158.4–183.5)	158.4 (154.3–175.2)	0.0051	<0.0001	175.2 (161.6–179.3)	154.3 (145.9–166.8)	154.3 (141.7–171.0)	0.0015	0.0027
GMI (%)	7.8 (7.4–8.1)	7.3 (7.1–7.7)	7.1 (7.0–7.5)	0.0052	<0.0001	7.5 (7.2–7.6)	7.0 (6.8–7.3)	7.0 (6.7–7.4)	0.0015	0.0027
CV (%)	39.4 ± 4.7	34.7 ± 5.9	33.6 ± 5.6	0.0064	0.00036	38.5 ± 5.7	34.0 ± 5.0	33.2 ± 5.1	0.0001	<0.0001
TIR_70–180_ (%)	54.0 (46.0–61.0)	66.0 (57.0–72)	66.0 (59.0–77.0)	0.0029	0.0001	61.0 (54.0–69.0)	73.0 (63.0–79.5)	72.0 (62.5–80.0)	<0.0001	<0.0001
TAR_180–250_ (%)	31.0 (24.5–33.0)	24.0 (18.0–28.0)	23.0 (18.0–28.0)	0.1219	0.0122	26.0 (22.5–29.0)	20.0 (16.0–24.5)	21.0 (16.0–26.0)	0.0067	0.0105
TAR_>250_ (%)	12.0 (7.0–19.3)	9.0 (5.0–13.0)	10.0 (3.0–13.0)	0.0539	0.0178	10.0 (6.8–12.5)	6.0 (3.0–9.0)	7.0 (3.0–10.0)	0.0193	0.0327
TBR_54–69_ (%)	1.0 (0.0–3.0)	1.0 (0.0–2.0)	1.0 (0.0–1.0)	0.3759	0.4141	2.0 (0.8–4.0)	1.0 (1.0–2.0)	1.0 (0.0–2.0)	0.0385	0.0078
TBR_<54_ (%)	0.0 (0–0)	0.0 (0–0)	0.0 (0–0)	0.4839	0.4839	0.0 (0–1.0)	0.0 (0–0.0)	0.0 (0–0.5)	0.5614	0.5614
TDD (IU/die)	-	43.2 (31.3–60.5)	42.7 (30.4–61.0)	-	0.9877	-	36.0 (30.3–51.8)	38.2 (29.5–47.9)	-	0.9997
TBD (%)	-	56.0 (51.0–65.0)	57.0 (52.0–62.0)	-	0.9983	-	55.0 (47.8–64.0)	56.0 (49.0–65.0)	-	0.9761
Total bolus (%)	-	44.0 (35.0–49.0)	43.0 (38.0–48.0)	-	0.9983	-	45.0 (36.0–52.3)	44.0 (35.0–51.0)	-	0.9761

Abbreviations. BMI, body mass index; CV, coefficient of variation; GMI, glucose management index; F, female; MDIs, multiple daily injections; TAR, time above range; TBD, total basal dose; TBR, time below range; TDD, total daily dose of insulin; TIR, time in range. The two-way ANOVA with Tukey multiple-comparison test was applied.

## Data Availability

The raw data supporting the conclusions of this article will be made available by the authors on request.

## Supplementary Materials

The following supporting information can be downloaded at: https://www.mdpi.com/article/10.3390/biomedicines14051136/s1, Figure S1. STROBE flow chart. Figure S2. CSII quality of life (CSII-QoL) questionnaire data stratified based on the previous MDI or OPD^®^ use. Two-way ANOVA with Tukey multiple comparisons test.
