# Supplementary material for: Transitioning to Omnipod 5®: Effectiveness, Safety, and Patient-Reported Outcomes of a Tubeless Automated Insulin Delivery System in Adults with Type 1 Diabetes Mellitus
Source: Biomedicines. 2026 May 17;14(5):1136. doi: 10.3390/biomedicines14051136 (PMC13205033; doi:10.3390/biomedicines14051136)
Supplement: Supplementary file 1 [file biomedicines-14-01136-s001.zip › biomedicines-4276375-supplementary.pdf]

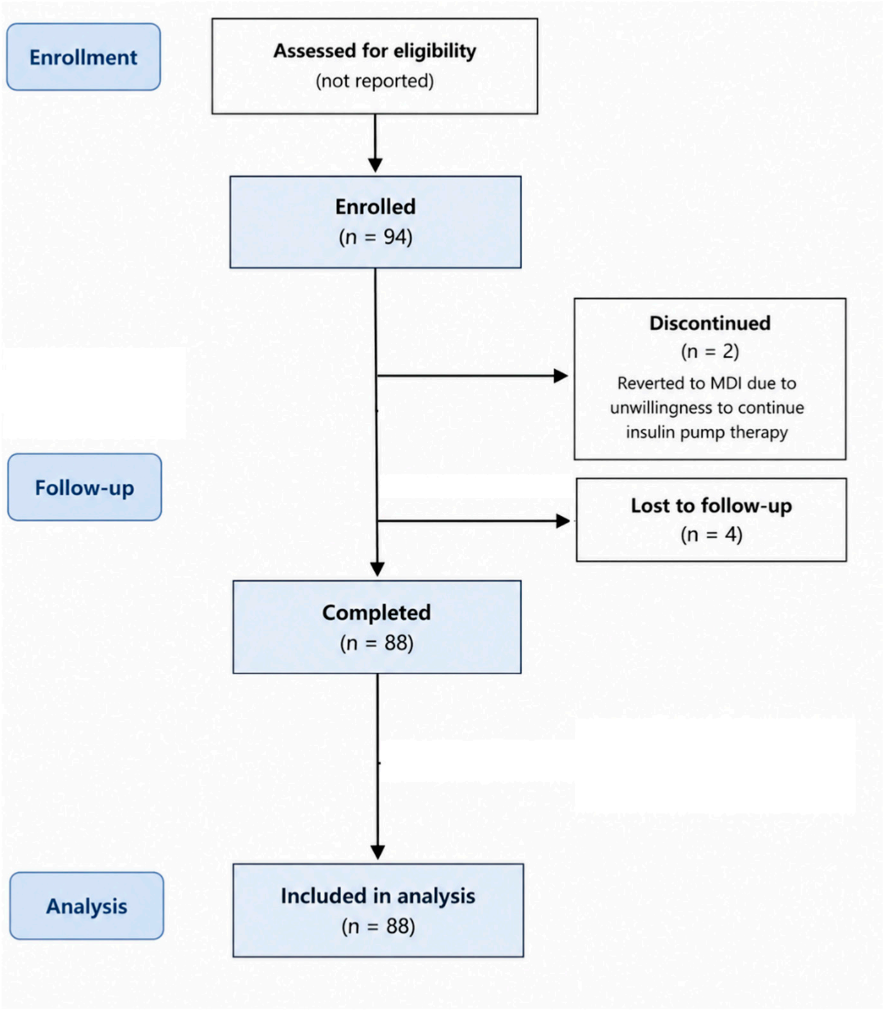

Supplementary Figure S1. STROBE flow chart.

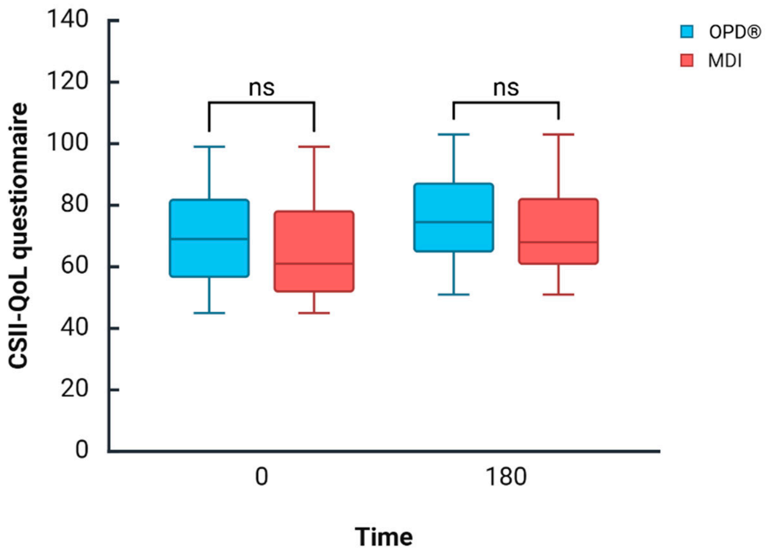

Supplementary Figure S2. CSII quality of life (CSII-QoL) questionnaire data stratified based on the previous MDI or OPD® use. Two-way ANOVA with Tukey multiple comparisons test.
